# Scaling digital models

**DOI:** 10.1038/s41598-026-36310-x

**Published:** 2026-01-21

**Authors:** Deniz Karanfil, Bahram Ravani

**Affiliations:** 1 Department of Mechanical and Aerospace Engineering, University of California, Davis, Davis, CA USA; 2 Advanced Highway Maintenance and Construction Technology (AHMCT) Research Center at the University of California, Davis, Davis, CA USA

**Keywords:** Engineering, Materials science, Mathematics and computing

## Abstract

The development of accurate digital models (DMs) for physical systems requires virtual representations that faithfully capture the underlying physics of the system or equipment being represented. Physics-based DMs provide reliable predictions only when accurate mathematical models of physical systems exist. When such models are incomplete or uncertain, experimental calibration can significantly improve model fidelity. However, in industries where systems or equipment exist in multiple sizes or configurations, performing experimental calibration for each variant can be prohibitively expensive and time-consuming. To address this challenge, this paper introduces a novel methodology and modular computational framework that leverages machine learning (ML) and dimensional analysis (DA) to enable scaling of DMs. The proposed approach allows calibration to be performed on a single representative system, with results scaled to other system sizes, whether from full-scale to reduced-scale prototypes or vice versa. Traditional applications of DA in this context often encounter difficulties due to distorted scaling factors. This work resolves these challenges by developing a consistent scaling framework tailored for DMs. The methodology is demonstrated by a case study in which a calibrated DM of a wheel loader is scaled between an industrial-size system and a miniaturized laboratory system.

**Supplementary Information:**

The online version contains supplementary material available at 10.1038/s41598-026-36310-x.

## Introduction

DMs are virtual representations of physical systems. When such models are utilized to develop digital twins, their behavior is continuously updated and refined through data obtained from their real-world counterparts^1^. These types of models are becoming increasingly integrated across a wide range of industries. Notably, in the construction sector, their adoption is gaining momentum^1–4^, which is a field that suffers significantly from low productivity in the execution of repetitive tasks^5–7^. In the context of construction vehicles, there has been previous research focusing on the development of physics-based models for systems such as excavators^8^, tower cranes^9^, and wheel loaders^10,11^. These DMs are valuable for estimating critical operational parameters, for example, forces acting on the bucket in excavation using wheel loaders. In recent work^11^, it has been shown that while physics-based virtual tools such as multibody dynamics (MBD) simulations can provide accurate models when there is no excavation; however, during excavation, estimation of excavation forces become very inaccurate. In such cases, it is shown^12^ that experimental calibration significantly improves the accuracy of the virtual model. Experimental calibration, however, can be costly and time consuming and for industries such as construction vehicles where there are multiple sizes of the same equipment, the process can become cumbersome and costly. In such situations, scaling techniques can reduce the burden so that experimental calibration can only be performed on a single size of a system and then the results are scaled to other sizes. Scaling techniques using DA have not yet been applied to the development of physics-based DMs. Conventional application of DA for scaling in this context, however, introduces several challenges due to the resulting distortion of scaling factors. This paper develops a computational tool using ML combined with DA to address these challenges.

The incorporation of ML into the proposed scaling framework is motivated by the highly nonlinear and nonuniform effects that arise from scaling distortions. Conventional regression techniques, or direct applications of similitude analysis, are often inadequate for capturing how these distorted scaling factors modify the underlying physical relationships among dimensionless parameters. Moreover, sole reliance on expert judgment to estimate the values of critical parameters at different scales becomes both costly and time intensive. This is the case particularly when the process has to be repeated at multiple scales, where distorted scaling factors introduce additional nonlinearities and significantly increase overall complexity. In contrast, a ML–based approach can approximate these complex nonlinear mappings directly from training data, enabling accurate estimation of scaled parameters even when similitude conditions are violated. By integrating ML with DA, the framework extends beyond ideal scaling regimes and remains effective in practical scenarios where systems from existing product lines serve as scaled representations of larger or smaller counterparts, despite significant deviations in scaling factors.

In this paper, neural networks are employed to estimate critical variables needed for calibration across different scales, even when distortions are present in the scaling factors. This is achieved through the involvement of the distortions and their effects on scaling through prediction factors^12^. In similitude analysis, the small-scale system is often referred to as the model and the larger-scale system as the prototype. A prediction factor refers to the ratio of the dimensionless numbers of the model and the prototype, which is not equal to the one due to scaling distortions^12^. The efficacy of the scaling framework developed here is demonstrated by applying it to scale bucket forces in excavation between two drastic sizes of wheel loader vehicles.

There seems to be no previously published work on application of similitude theory and DA to scale calibrated DMs. In other applications where DA and similitude theory have been used, no existing framework has been developed for scaling parameters between pairs of systems with distorted scaling factors that violate similitude. Furthermore, existing approaches cannot capture the nonlinear relationships introduced by such distortions in system pairs from real industrial product lines (as discussed in the section on State of the Art). This paper addresses these gaps by developing a ML-based scaling framework that allows accurate transfer of calibration data between DMs at vastly different scales, even when similitude is violated.

## State of the art

To scale a model, it is essential to utilize dimensionless numbers. The foundational ideas of physical dimensions and DA were first introduced by Joseph Fourier and Lord Rayleigh, respectively^13–16^. Scaling laws, which are relationships derived from dimensionless numbers, provide a systematic framework for predicting system behavior across different scales. Scaling facilitates the design and analysis of complex and large-scale physical systems by reducing reliance on extensive computational resources or large-scale experimental setups that increase costs and limit feasibility^12,17^. DA is vital for developing scaling models because it reduces the complexity of the problem by reducing the number of parameters involved in it^18^. The Buckingham Pi theorem^19^ is a fundamental tool in DA, and it states that systems can be modeled with a reduced number of parameters using dimensionless numbers. The theorem also facilitates the systematic derivation of the dimensionless numbers of physical systems, which makes the analyses of systems more robust when it comes to experimental design, data correlation, and interpretation of the results. It also enables the derivation of scaling laws for systems where similitude requirements hold true^20^.

Previous studies in similitude analysis have explored methodologies and algorithms capable of automatically generating dimensionless numbers via the Buckingham Pi theorem^21^. Although the Buckingham Pi theorem can be employed to derive these terms, using them directly to scale physical parameters poses critical limitations. Namely, the application of the theorem does not result in unique solutions (dimensionless numbers)^12^. Furthermore, the theorem on its own is inadequate to discern which dimensionless terms hold higher physical significance^22^. The terms with lower physical significance may offer limited utility of the scaling process if they do not exhibit strong correlations with the parameters intended to scale. Xie et al.^23^ have focused on identifying the significant dimensionless numbers from data by employing ML and data-driven analysis. Although more significant dimensionless numbers can be identified, a notable gap in the literature remains on the impact of distorted scaling factors on how the parameters of interest scale. Established scaling laws can break down even with minor distortions to the scaling factors, as these distortions alter the underlying relationships between the dimensionless numbers. While there have been previous studies where the conventional Pi theorem–based scaling approach was employed for complex mechanical systems (see, for example, Zhao et al.^24^), the application of scaling laws necessitates that no distortion is present in any of the scaling factors between different scales of a system^25,26^. In fact, existing regression-based or Pi-theorem based approaches cannot capture the nonlinear relationships introduced by distortions. This paper develops a methodology by combining ML with DA to address this problem. In a way this paper addresses two gaps in existing literature. The first is application of similitude theory and DA to DMs and second developing a method that would allow parameter scaling between systems that violate similitude conditions due to distorted scaling factors. The results are demonstrated through a case study using DMs of two sizes of wheel loader vehicles.

Previous literature in modeling the relationship between the dimensionless terms so that the parameter of interest can be scaled includes the work of Kasprzak et al.^27^. Regression methods and a variety of modeling assumptions are often employed to capture relationships among dimensionless terms^28^. For instance, the algorithms proposed by Mendez and Ordonez utilize power law formulations in this context^29^. However, such power law formulation only applies to a limited class of problems. In the context of scaling calibrated DMs, the effect of distortions must also be included in the model. Neural networks often offer higher versatility compared to traditional regression methods when it comes to complex and highly nonlinear relationships^30^ and that is why they are used here.

## Methods

### Determination of the number of input parameters in the scaling framework

DA typically uses the Buckingham Pi theorem^19^ to determine the number of dimensionless numbers required to express the model of the physical system in a dimensionless form. The dimensionless form results in a reduced number of parameters, which is expressed by Eq. (1) ^19^.

1 $$n = p - f$$

where $p$ denotes the number of parameters in the original form of a physical system, $n$ represents the number of parameters in the dimensionless form, and $f$ corresponds to the number of fundamental units involved. Fundamental units, also referred to as fundamental dimensions, are the units which cannot be expressed in terms of other units^12^. The fundamental units of a system are determined by the energy domains (mechanical, electrical, etc.) involved in the physical system under consideration. How many fundamental units a system has ($f$) can be found as shown in Eq. (2) ^31^.

2 $$f = \mathop \sum \limits_{1}^{i} \beta_{i} - \mathop \sum \limits_{2}^{i} \xi_{1i}$$

where $i$ refers to the number of energy domains present in the system. ${\beta }_{i}$ denotes how many fundamental units there are within the domain $i$. ${\xi }_{1i}$ represents the number of fundamental units that domains 1 and $i$ have in common.

### Identification of parameters influencing the scaled quantity

Once the number of input parameters is established, the next step in DA is to identify the parameters that influence the target quantity to be scaled, based on the governing dynamics of the system. The selected parameters are reformulated into dimensionless groups using DA. The target quantity is incorporated into one of the dimensionless terms, which is designated as the output of the model, to enable scaling. A Buckingham Pi theorem-based computer program is employed to generate a large number of dimensionless numbers based on the parameters that would be included in the model. These dimensionless numbers then go through another computer program, which was developed to incorporate a correlation-based filtering mechanism so that a set of dimensionless terms that exhibit strong correlation with the parameter being scaled can be identified. The overall correlation strength of each set was evaluated based on the Pearson correlation coefficient^32,33^ between the target parameter and each individual dimensionless term, followed by calculating the root-mean-square (RMS) of these individual correlation values.

### Inclusion of distortion effects on scaling

In an ideal scenario, where scaling can be achieved by a uniform scaling factor, the scaling laws can be defined in the following format^12^:

3 $$\pi_{n}^{m} = \pi_{n}^{p}$$

where the dimensionless numbers of one model are denoted with ${{\pi }_{n}}^{m}$ and those of the prototype are represented by ${{\pi }_{n}}^{p}$. When distorted scaling factors are involved, the scaling laws will instead adhere to the relationship described in Eq. (4) below:

4 $$\pi_{n}^{m} = \delta_{n} \pi_{n}^{p}$$

For the dimensionless term with the target parameter:

5 $$\pi_{1}^{m} = \delta_{1} \pi_{1}^{p}$$

In Eq. (5), ${\delta }_{1}$ is referred to as the prediction factor. This factor is a function of the distortions and the remaining dimensionless numbers^12^. Here, we develop a compact formulation to model ${\delta }_{1}$ in a manner that includes the effects of both the remaining dimensionless numbers and the scaling distortions. This relationship is expressed as shown in Eq. (6):

6 $$\delta_{1} = \delta_{1} \left( {d_{2} ,d_{3} ,d_{4} \ldots { }d_{n} } \right)$$

where each distortion term ${d}_{n}$ is defined by the ratio of the dimensionless number of the model to that of the prototype:

7 $$d_{n} = \frac{{\pi_{n}^{m} }}{{\pi_{n}^{p} }}$$

Since ${{\pi }_{1}}^{m}$ is a known quantity in Eq. (5), which is obtained from the equipment implemented on the small scale model, ${{\pi }_{1}}^{p}$ can be evaluated using the prediction factor ${\delta }_{1}$ estimated by the scaling model. Once ${{\pi }_{1}}^{p}$ is determined, the scaled parameter for the prototype, which is costly and challenging to measure directly on the large-scale prototype, can be estimated by substituting the known, physical parameters as follows:

8 $$\delta_{1} = \frac{{\pi_{1}^{m} }}{{\pi_{1}^{p} }}$$

Once the target parameter is accurately scaled by the model, the instrumented unit in a product line can be leveraged to establish the DM of a different unit in the same product line with vastly distorted scaling factors without the need for additional instrumentation and calibration.

### Training process of the scaling model

ML models are employed to model the relationships between the distortion terms so that the target parameters can be accurately scaled. The ML model is trained using data from the smaller scale calibrated counterpart. Once trained, the framework can be applied to predict scaled parameters for new system configurations, eliminating the need for additional experimental calibration. The methodology is formulated in terms of governing physical parameters, dimensionless groups, and distortion effects.

## Case study of a wheel loader digital model

### Calibrated digital model of a wheel loader

**Fig. 1:**
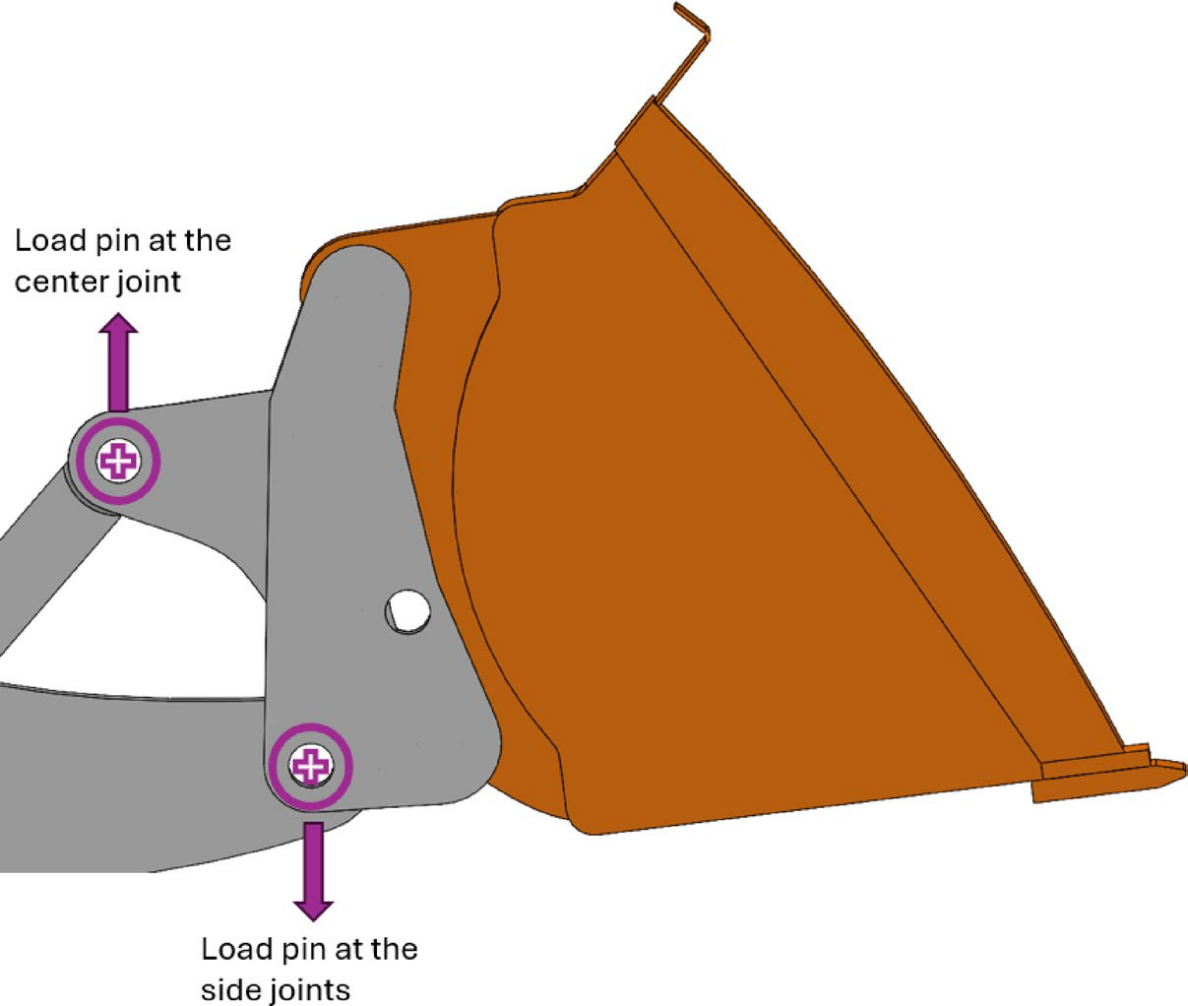
Locations of the hinges where the load pins measuring the forces on the bucket were mounted, shown in the side view of the bucket CAD model.

The DM employed in this study is a physics-based DM of a commercial wheel loader. Its development involved the integration of a high-fidelity computer-aided design (CAD) model into physics-based MBD simulations using the software Algoryx Dynamics^34,35^. The main purpose of this DM is to accurately estimate the forces acting on the bucket of the wheel loader during excavation of a pile of soil or rocks, which has been used for tasks such as soil parameter estimation^36^ and powertrain control^37^. This DM was calibrated using real-world experimental data gathered by instrumenting the commercial wheel loader with a wide array of sensors including an inclinometer, a quadrature encoder, pressure transducers, and load pins mounted at the joints between the base of the bucket and the remaining links to measure forces acting on the bucket, as illustrated in Fig. 1^11^. The idea here is to scale this model to develop DMs of other wheel loader of different sizes without repeating the calibration process. In this fashion, much of the cost and effort in instrumentation and experimentation for calibration of other sizes of the wheel loader product line can be eliminated.

A commercial wheel loader was instrumented with load pins in the side and the center joints of its bucket (see Fig. 1) to obtain experimental estimates of the excavation forces. If estimates of excavation forces from an instrumented wheel loader can be scaled to other sizes of such vehicles without the need to instrument them, then scaling provides much savings in cost and effort. This is the hypothesis which is proved in this paper.

**Fig. 2:**
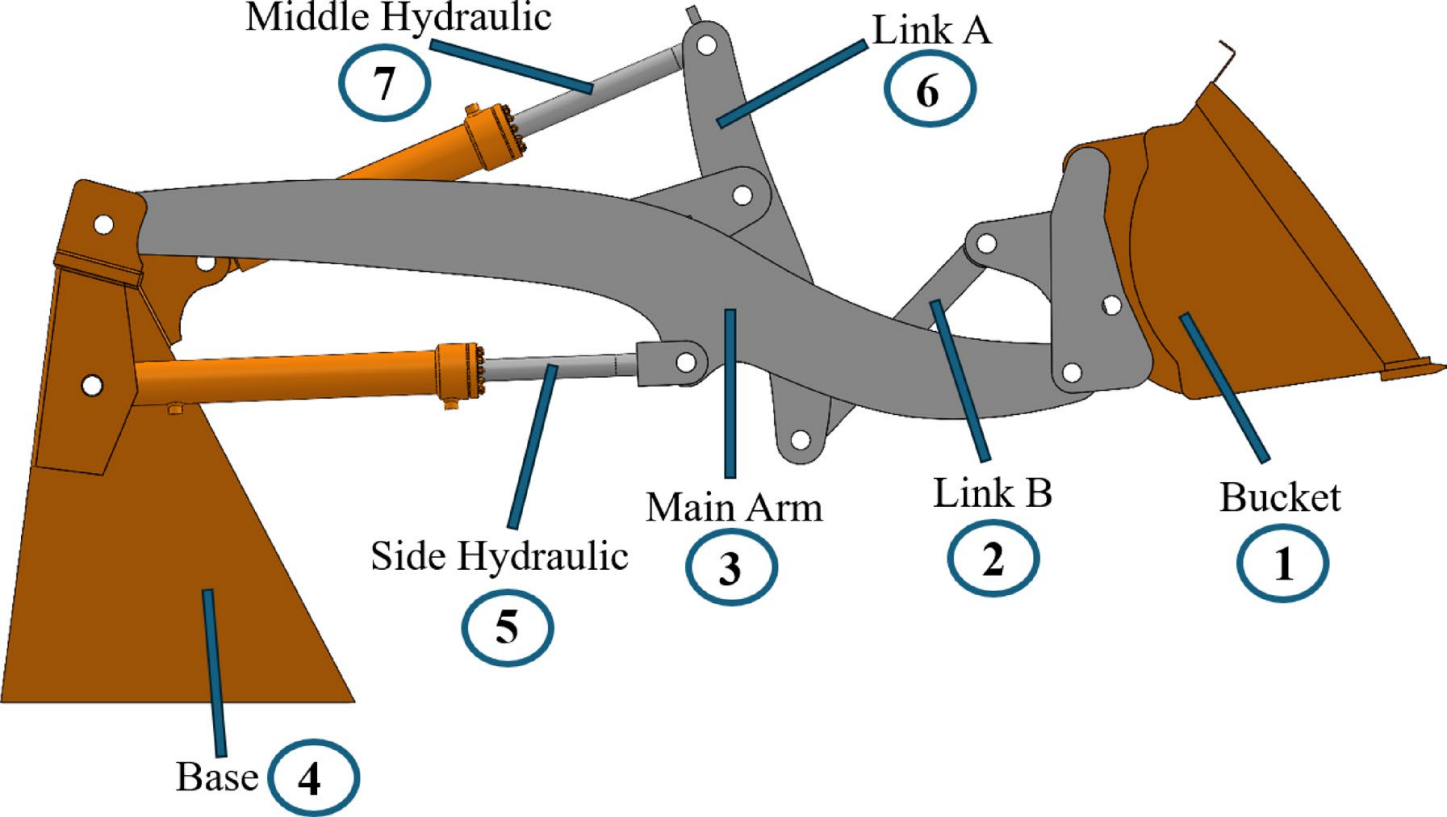
Names and the index numbers of the mechanism components shown in the technical drawing of the wheel loader mechanism CAD model of the ER12.

In scaling, it is essential to identify which parameters influence the target parameter being scaled, based on the physics of the system. The next section explains this process in detail. The bucket lifting mechanism of the commercial wheel loader used is shown in Fig. 2.

The bucket lift mechanism of a wheel loader has hydraulic and mechanical systems, which share all three of their fundamental units (kilograms, meters, and seconds representing mass, length, and time, respectively). Applying Eq. (2) to this system leads to the following equations:

9 $$f = \beta_{1} + \beta_{2} - \xi_{12}$$

10 $$f = \beta_{1} = \beta_{2} = \xi = 3$$

Thus, the system can be expressed with $n=p-3$ parameters in a dimensionless form, which corresponds to the number of inputs required for the parameter scaling process.

**Fig. 3:**
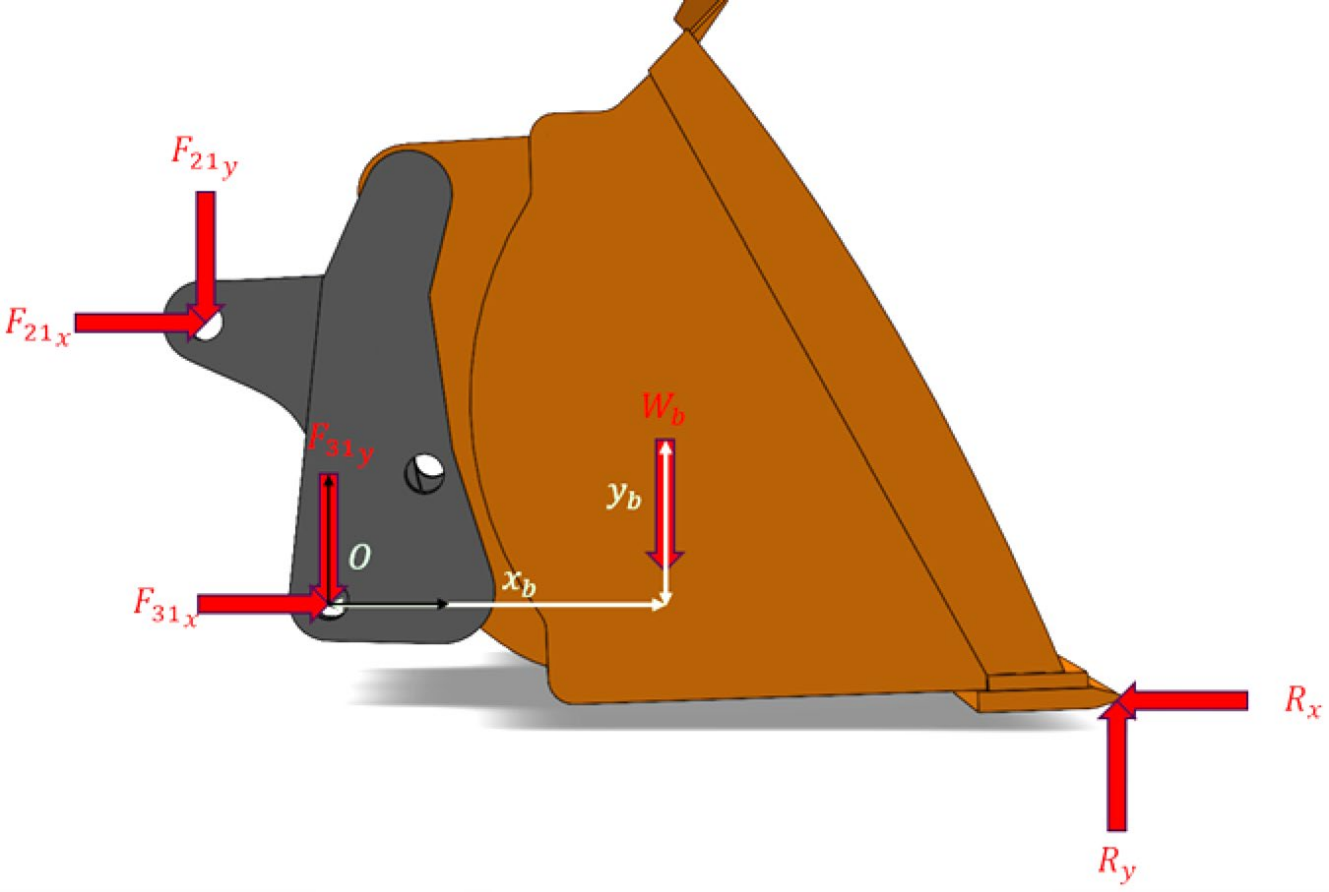
Free body diagram of the bucket, showing the components of the excavation reaction force acting on the bucket tip (${{\boldsymbol{R}}}_{{\boldsymbol{x}}}$ and ${{\boldsymbol{R}}}_{{\boldsymbol{y}}}$), combined weight of the bucket and the load it is carrying (${{\boldsymbol{W}}}_{{\boldsymbol{b}}}$), and the joint reaction forces at the connecting joint with Link B (${{{\boldsymbol{F}}}_{21}}_{{\boldsymbol{x}}},{{{\boldsymbol{F}}}_{21}}_{{\boldsymbol{y}}}$), as well as with the Main Arm (${{{\boldsymbol{F}}}_{31}}_{{\boldsymbol{x}}},\boldsymbol{ }{{{\boldsymbol{F}}}_{31}}_{{\boldsymbol{y}}}$).

**Fig. 4:**
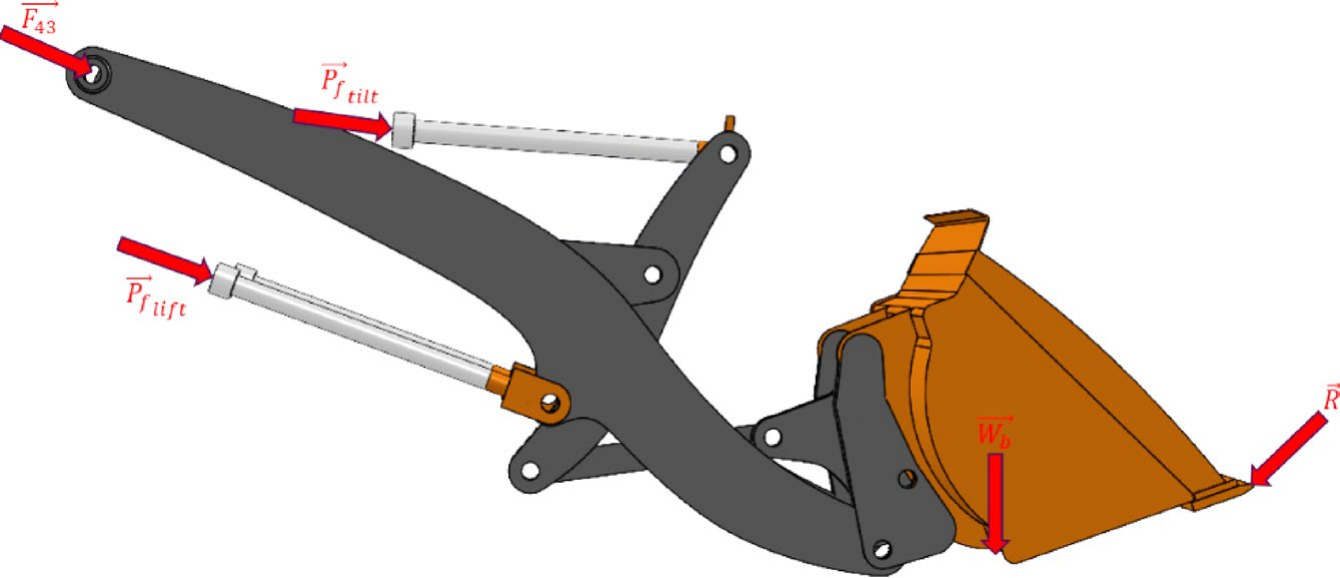
Free body diagram of the overall mechanism, showing the excavation reaction force acting on the bucket tip ($\overrightarrow{{\boldsymbol{R}}}$), combined weight of the bucket and the load it is carrying ($\overrightarrow{{{\boldsymbol{W}}}_{{\boldsymbol{b}}}}$), the forces from the hydraulic cylinders tilting the bucket (${\overrightarrow{{{\boldsymbol{P}}}_{{\boldsymbol{f}}}}}_{{\boldsymbol{t}}{\boldsymbol{i}}{\boldsymbol{l}}{\boldsymbol{t}}}$) and lifting the mechanism (${\overrightarrow{{{\boldsymbol{P}}}_{{\boldsymbol{f}}}}}_{{\boldsymbol{l}}{\boldsymbol{i}}{\boldsymbol{f}}{\boldsymbol{t}}}$), and the joint reaction forces at the connecting links with the base of the vehicle ($\overrightarrow{{{\boldsymbol{F}}}_{43}}$). In the training data gathered through MBD-based DM, weights of all components are included; however, the scaling models consider only the combined weight of the bucket and its load, as these dominate the dynamic behavior of the system. Note that the weights of the links are neglected.

In order to select the set of $n$ dimensionless numbers, the next step involves identifying which parameters have influence over the scaled parameter ($\text{force at the central load pin location}$) based on the underlying physics (in this case, dynamics) of the system. Figures 3 and 4 demonstrate the free body diagrams of the bucket and the overall mechanism. ${F}_{ab}$ represents the force at the joint connecting the components numbered $a$ and $b$, whereas ${{F}_{ab}}_{x}$ and ${{F}_{ab}}_{y}$ represent the $x$ and $y$ components of ${F}_{ab}$, respectively.

Let $R,$ ${I}_{O},$ ${m}_{1}$, ${{\boldsymbol{\upalpha}}}_{1}$, and ${\mathbf{a}}_{1}$ denote, respectively, the excavation reaction force acting on the bucket, the mass moment of inertia about point $O$, the mass, angular acceleration, and the linear acceleration at the center of mass. The following equations of motion are obtained from the bucket’s free-body diagram:

11 $$\sum {\mathrm{F}} = m_{1} {\mathrm{a}}_{1}$$

12 $$\sum {\mathrm{F}} = {\mathrm{F}}_{21} + {\mathrm{F}}_{31}$$

13 $$\sum {\mathrm{M}}_{O} = I_{O} {\upalpha }_{1}$$

14 $$\sum {\mathrm{M}}_{O} = {\mathrm{r}}_{21} \times {\mathrm{F}}_{21} + {\mathrm{r}}_{{\mathrm{W}}} \times {\mathrm{W}} + {\mathrm{r}}_{{\mathrm{R}}} \times {\mathrm{R}}$$

where ${\mathbf{r}}_{21}$, ${\mathbf{r}}_{\mathbf{W}}$, and ${\mathbf{r}}_{\mathbf{R}}$ denote the position vectors corresponding to ${\mathbf{F}}_{21}$, the weight being carried $\mathbf{W}$, and the ground reaction $\mathbf{R}$.

Let ${{\mathbf{P}}_{\mathbf{f}}}_{\mathbf{t}\mathbf{i}\mathbf{l}\mathbf{t}}$ and ${{\mathbf{P}}_{\mathbf{f}}}_{\mathbf{l}\mathbf{i}\mathbf{f}\mathbf{t}}$ denote the forces generated by the pressures on the hydraulic cylinders responsible for tilting and lifting the buckets. Then, for the free body diagram of the overall mechanism:

15 $$\sum {\mathrm{F}} = {\mathrm{F}}_{43} + {\mathrm{R}} + {\mathrm{W}} + {\mathrm{P}}_{{{\mathrm{f}}_{{{\mathrm{lift}}}} }} + {\mathrm{P}}_{{{\mathrm{f}}_{{{\mathrm{lift}}}} }}$$

**Fig. 5:**
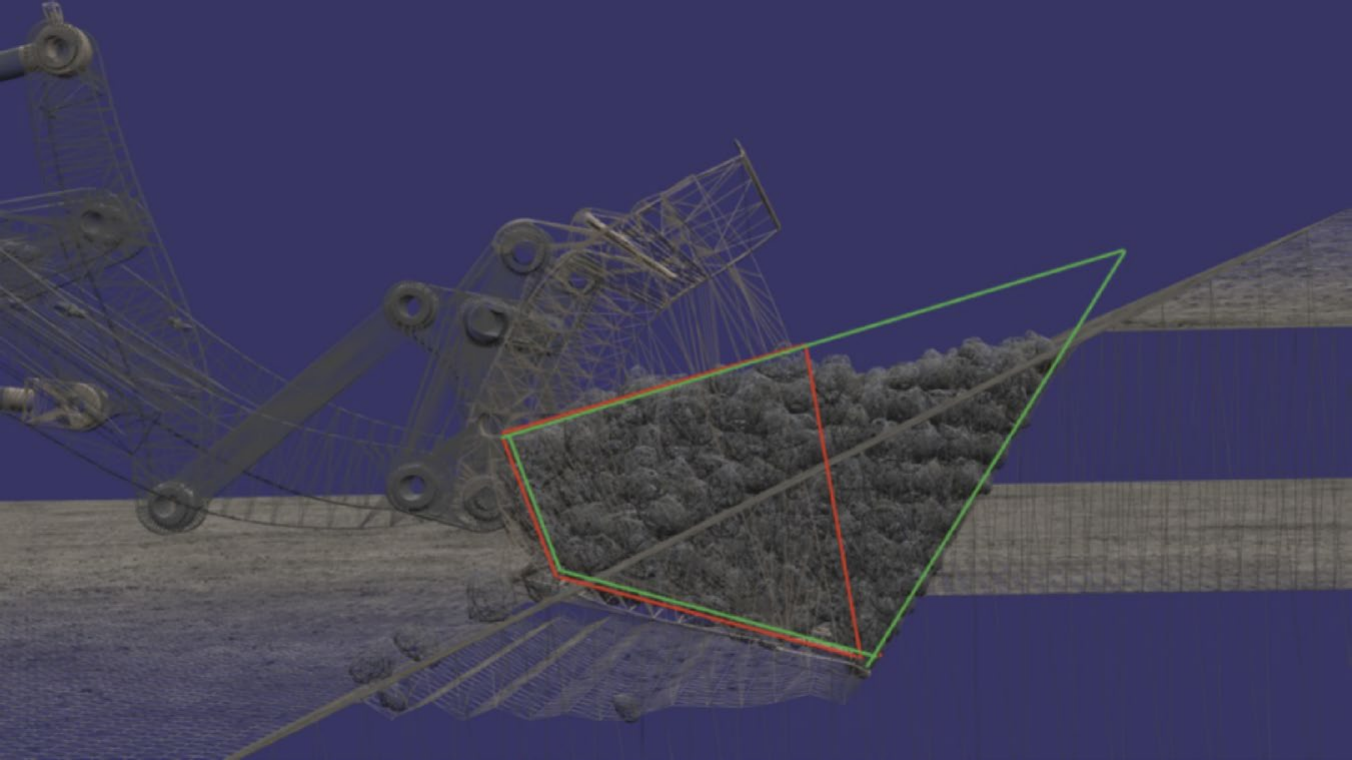
The green area encompassing all terrain bodies generated in a given time, and the red area encompassing the material inside the bucket.

As illustrated in Fig. 5, while generating the training data of the scaling model, the simulation environment of the DM was modified so that the bucket and the load that it carries were treated as a combined dynamic body. In this configuration, both the mass and the center of mass location varied depending on the amount of load material loaded from the pile.

Based on the dynamics of the system, the following set of parameters in Eq. (16) was chosen to be included in the model that is designed to scale the target parameter, ${F}_{21}$ (magnitude of the force at the central joint load pin), which is being scaled under the influence of distorted scaling factors.

16 $$F_{21} = F_{21} \left( {F_{31} ,P_{{f_{tilt} }} ,P_{{f_{lift} }} ,x_{b} ,y_{b} ,m_{b} ,a_{1} ,\alpha_{1} } \right)$$

In this last equation, ${x}_{b},{y}_{b}$, and ${m}_{b}$ represent the center-of-mass coordinates and the dynamic mass of the combined body (bucket and load), respectively, in the rotating coordinate system attached to point $O$ (as shown in Fig. 3). On the other hand, ${F}_{31},{{P}_{f}}_{tilt},{{P}_{f}}_{lift},{a}_{1}$ and ${\alpha }_{1}$ denote the magnitudes of ${\mathbf{F}}_{31},{{\mathbf{P}}_{\mathbf{f}}}_{\mathbf{t}\mathbf{i}\mathbf{l}\mathbf{t}}$, ${{\mathbf{P}}_{\mathbf{f}}}_{\mathbf{l}\mathbf{i}\mathbf{f}\mathbf{t}}$, ${\mathbf{a}}_{1}$, and ${{\boldsymbol{\upalpha}}}_{1}$.

Using Eq. (1), it can be observed that Eq. (16), which includes 9 parameters, can be reformulated in a dimensionless form involving only 6 parameters, as follows:

17 $$n = p - f = 9 - 3 = 6$$

The dimensionless form of Eq. (16) is expressed as shown in Eq. (18).

18 $$\pi_{1} = F\left( {\pi_{2} ,\pi_{3} ,\pi_{4} ,\pi_{5} ,\pi_{6} } \right)$$

where ${\pi }_{\mathrm{n}}$ denotes dimensionless terms derived from parameters in the original form of Eq. (16). The term ${\pi }_{1}$ is the output of the model, and it is deliberately chosen to include the target parameter, ${F}_{21}$, so that its scaling can be facilitated.

19 $$\pi_{1} = \frac{{F_{21} }}{{F_{31} }}$$

Since this system can be expressed using 6 dimensionless parameters, the prediction factor incorporating the target parameter being scaled can be modeled as follows:

20 $$\delta_{1} = \delta_{1} \left( {d_{2} ,d_{3} ,d_{4} ,d_{5} ,d_{6} } \right)$$

Once ${{\pi }_{1}}^{p}$ is determined using ${{\pi }_{1}}^{m}$ from the commercial wheel loader and ${\delta }_{1}$ from the scaling model, the scaled parameter ${{F}_{21}}^{p}$, can be estimated as follows:

21 $$\pi_{1}^{p} = \frac{{F_{21}^{p} }}{{F_{31}^{p} }}$$

22 $$F_{21}^{p} = F_{31}^{p} \left( {\frac{{\pi_{1}^{m} }}{{\delta_{1} }}} \right)$$

Once the target parameter is accurately scaled by the model, the instrumented/calibrated DM of a unit in a product line can be leveraged to establish the DM of a different unit in the same product line with vastly distorted scaling factors without the need for additional instrumentation and calibration.

A total of three wheel loaders were considered within the scope of this study. The calibrated DM of a medium-sized 3300 kg EVERUN ER12 wheel loader, as well as the simulations of a roughly 26,000 kg Komatsu WA475 wheel loader, were utilized to generate the training data of the model and to verify it. The third wheel loader was chosen as a miniature 11 kg Kabolite wheel loader, which was employed to validate the accuracy of the trained models under extreme scale differences with large degrees of scaling distortions. In each simulation, wheel loaders carry out a similar sequence of standardized actions where they move in a straight line, lower the bucket to collect material, and then pull back after completing the loading action. The evaluated parameters are recorded from the start to the end of the operation and incorporated into the training data. Three distinct models were trained to scale the target parameter. The first one employed feed-forward neural networks (FFNN)^38^ utilizing the parameter set from Eq. (16). The second one was a Gated Recurrent Unit (GRU)-based^39^ recurrent neural network (RNN)^40^ with the same set of parameters. The last one also utilized RNNs but used an alternative parameter set, detailed below.

23 $$F_{21} = F_{21} \left( {F_{31} ,P_{{f_{tilt} }} ,P_{{f_{lift} }} ,x_{b} ,y_{b} ,m_{b} ,V_{1} , w_{1} } \right)$$

In both the FFNN- and RNN-based models, the input–output relationships were structured based on Eq. (20). The distortion terms (${d}_{n}$) were set as the inputs and the prediction factor for the parameter of interest was designated as the single output. For FFNN-based model, this relationship is represented as a static mapping between input vectors and the targeted output, which captures nonlinear dependencies through multiple connected layers. More details of the ML methods used are discussed in the Results section. Unlike the FFNN-based model, the temporal sequence of the training data plays a critical role in capturing dynamic behavior for the models using RNNs. To preserve these temporal dependencies inherent in the simulation, the training data were organized into ordered sequences that reflect how the events progress within each simulation run. Each sequence was constructed such that the order in which data points were fed into the model corresponded to how the timeline progressed in the simulations. The dimensionless term associated with the parameter that is intended to be scaled is set as the model’s output (${{\pi }_{1}}^{m}/{{\pi }_{1}}^{p}$, or $({{F}_{21}}^{m}{{F}_{31}}^{p})/({{F}_{31}}^{m}{{F}_{21}}^{p})$), while the rest of the dimensionless terms serve as inputs. The model whose results are scaled is designated randomly as one row from the training dataset corresponding to the DM of the ER12 medium-size wheel loader. Each modeling approach was evaluated based on its accuracy in scaling the target parameter. Furthermore, hyperparameter optimization was utilized to fine-tune key architectural and training parameters, including the number of hidden layers^41^, the number of neurons per hidden layer^42^, the dropout rate^43^, and the learning rate^44^. Hyperparameter optimization results and further details of the network architecture are provided in the Results section.

In a practical setting, scaling would typically be performed from the small-scale model to the large-scale prototype. However, the developed scaling framework can perform the scaling process in both directions. Scaling aspect is also not unique to a specific pair of systems. Once trained, the model can predict parameters of interest across and between all three scales of wheel loaders considered in this study, which are the miniature, medium-sized, and large wheel loaders. The miniature wheel loader was utilized for validation. This was done to demonstrate the framework’s ability to generalize under large scale differences with significant scaling distortions, as no data from the miniature loader were included in the training process of the framework. This bidirectional functionality and cross-scale adaptability emphasize the general applicability of the framework to diverse product lines with varying scales.

## Results

### Assessing the accuracy of the scaling models

**Fig. 6:**
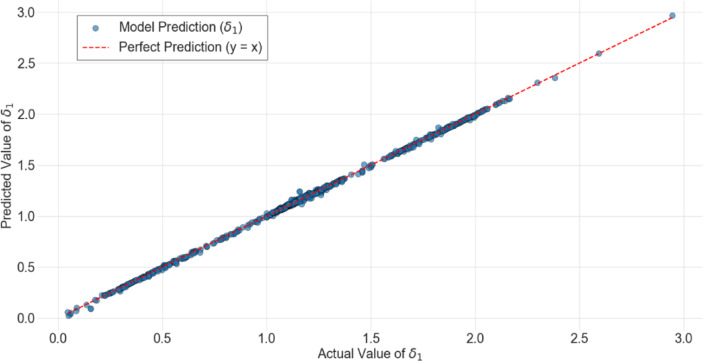
Values of the prediction factor ${{\boldsymbol{\delta}}}_{1}$ predicted by the FFNN-based model plotted against the actual values, with the y = x trendline representing perfect accuracy in scaling.

**Fig. 7:**
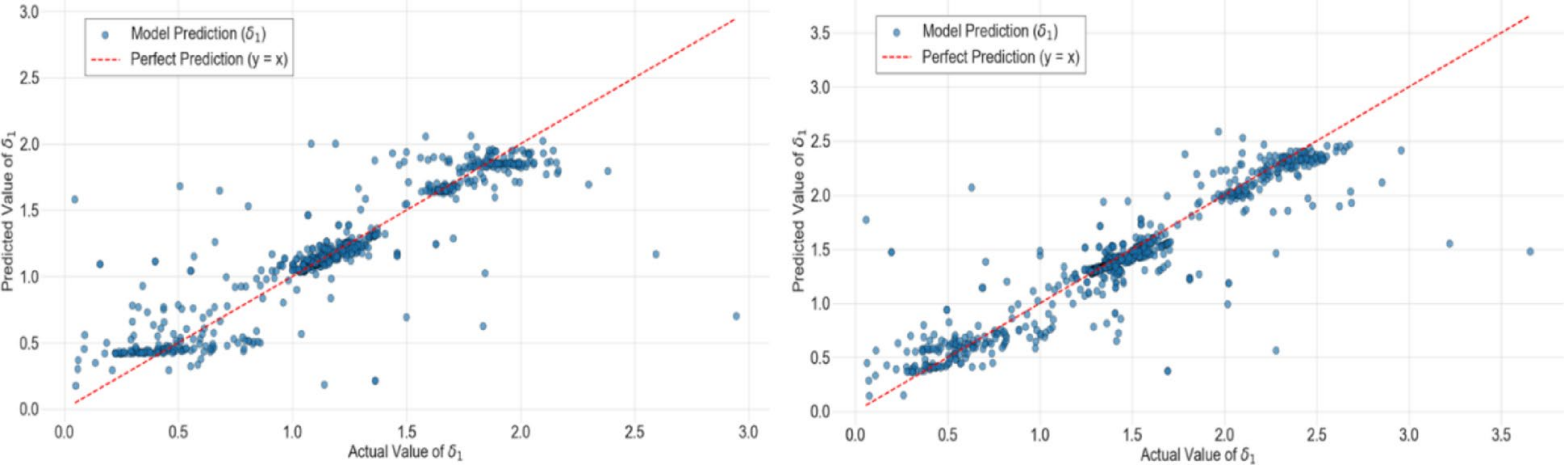
Values of the prediction factor ${{\boldsymbol{\delta}}}_{1}$ predicted by the RNN-based models plotted against the actual values, with the y = x trendline representing perfect accuracy in scaling.

Figures 6 and 7 illustrate the performance of the models trained on the same dataset, evaluated using the coefficient of determination (${R}^{2}$)^45^. Data for the medium-sized and large wheel loaders are used to train these models and evaluate their performance. Data for the miniature wheel loader are not included in the training set of these models and is used only for validation at an extreme scale difference, as discussed further in the next section on validation. In these figures, the values that models predict for ${\delta }_{1}$, which gives the scaled values of the parameter of interest as explained in Eqs. (19)–(22), are plotted against the real values of ${\delta }_{1}$. The $y=x$ lines represent perfect alignment, serving as a reference for evaluating the model’s performance. Figure 6 shows the results of the FFNN-based model employing the set of parameters from Eq. (16). This model exhibited the best performance with an ${R}^{2}$ value of 0.99. An ${R}^{2}$ value of 1.00 represents perfect agreement between the real values of the parameter and the ones estimated by the scaling model. This high value of ${R}^{2}$ indicates that the proposed ML-based scaling framework successfully captures the nonlinear dependencies introduced by distorted scaling factors. Based on the hyperparameter optimization, the (FFNN)-based model used to generate this figure consisted of two hidden layers of 96 neurons each with ReLU activation functions, followed by a single linear output neuron, resulting in a total of 9985 trainable parameters. The model received five dimensionless input features (${\delta }_{2}$ to ${\delta }_{6}$) and produced one output (${\delta }_{1}$). All input and output variables were standardized to zero mean and unit variance using z-score normalization based on the training set. The network was trained using the Adam optimizer (learning rate = 0.0005) and mean-squared-error (MSE) loss function, for 100 epochs with a batch size of 16, without dropout regularization.

Figure 7 illustrates the performance of the GRU-RNN-based models, where the one incorporating the set from Eq. (23) is shown on the right and the one using the set from Eq. (16) is shown on the left.

For the model based on Eq. (23), the input data were organized into ordered sliding-window sequences of five time-steps, each containing five features. Sequences were generated within each simulation segment to preserve the correct temporal order and to avoid data leakage, with the target corresponding to the value at the next step. The network consisted of four stacked GRU layers, each containing eight units. The first three layers were configured to return full sequences, while the final GRU layer output only the last state. All GRU layers used tanh activation for the output and sigmoid for the recurrent connections. The layers were stateless, used the reset after parameter, and had no dropout or recurrent dropout applied. A single linear dense layer produced the output, giving the model a total of 1665 trainable parameters. Before training, both input and target data were normalized using z-score standardization based on the training dataset. The network was trained using the Adam optimizer with a learning rate set to 0.0005 and MSE as the loss function. Training was performed for 100 epochs, with a batch size set to 16. For the model based on Eq. (16), the data preprocessing and sequence construction steps were identical to those of the model using Eq. (23), with five-time steps with five features per sequence, the same normalization, and the same training procedure. The network differed in its architecture and hyperparameters: it comprised three stacked GRU layers with sixteen units each, where the first two layers returned full sequences and the last layer returned only the final state. Each GRU layer was followed by a dropout layer with a rate of 0.2 to improve generalization. The GRUs used tanh and sigmoid activations for the output and recurrent connections, respectively, were stateless, and employed the reset after configuration. A single linear dense layer produced the final output, giving a total of 4385 trainable parameters. The model was trained using the Adam optimizer with a learning rate set to 0.01. MSE was set as the loss function. Training was again performed for 100 epochs, with batch size set to 16. These hyperparameters were determined through the same grid-search process applied to the model using Eq. (23). These two models yielded ${R}^{2}$ values of 0.85 and 0.81, respectively, which indicates reduced predictive performance compared to the FFNN-based model.

**Fig. 8:**
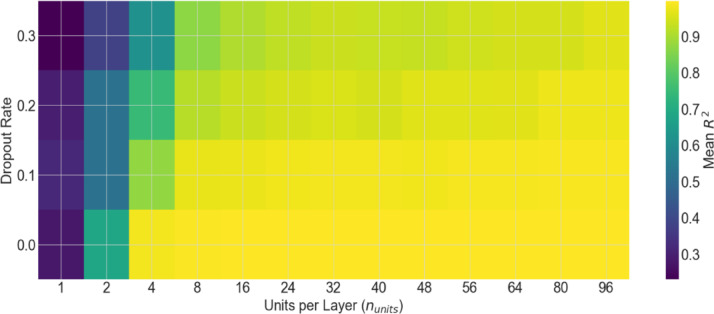
Visualization of the grid search method, illustrating the impact of hidden layer width and dropout rate on model performance, measured by the mean ${{\boldsymbol{R}}}^{2}$.

**Fig. 9:**
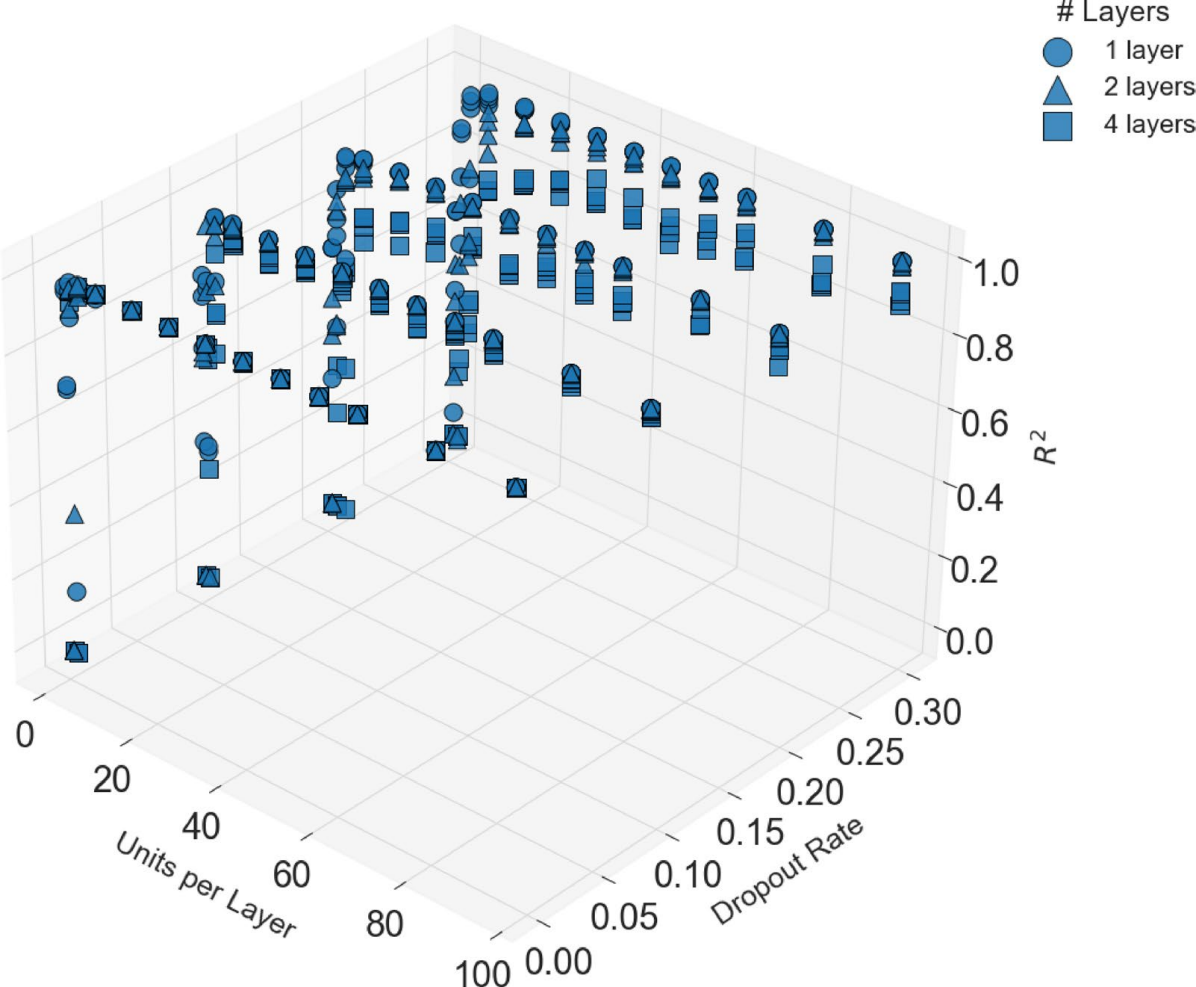
Visualization of the grid search method, illustrating the hyperparameter landscape with the effect of hidden layer width, dropout rate, learning rate (color scale), and number of layers (marker shape) on model performance.

Figures 8 and 9 present the grid search process^46,47^ conducted to optimize the hyperparameters of the FFNN-based model. Figure 8 presents a two-dimensional heat map which depicts model performance as a function of the number of units per layer and dropout rate. The color in this figure indicates the mean ${R}^{2}$ value. As can be seen from the figure, configurations with narrow layers and high dropout rates exhibit significantly degraded performance, whereas lower dropout rates combined with moderate to large numbers of units per layer achieved the best performance. Figure 9 demonstrates a scatter plot of the hyperparameter landscape where the number of units per layer, dropout rate, and the resulting ${R}^{2}$ represent the three axes. Marker shape denotes the number of hidden layers.

### Validation of the scaling model accuracy on a miniature wheel loader

**Fig. 10:**
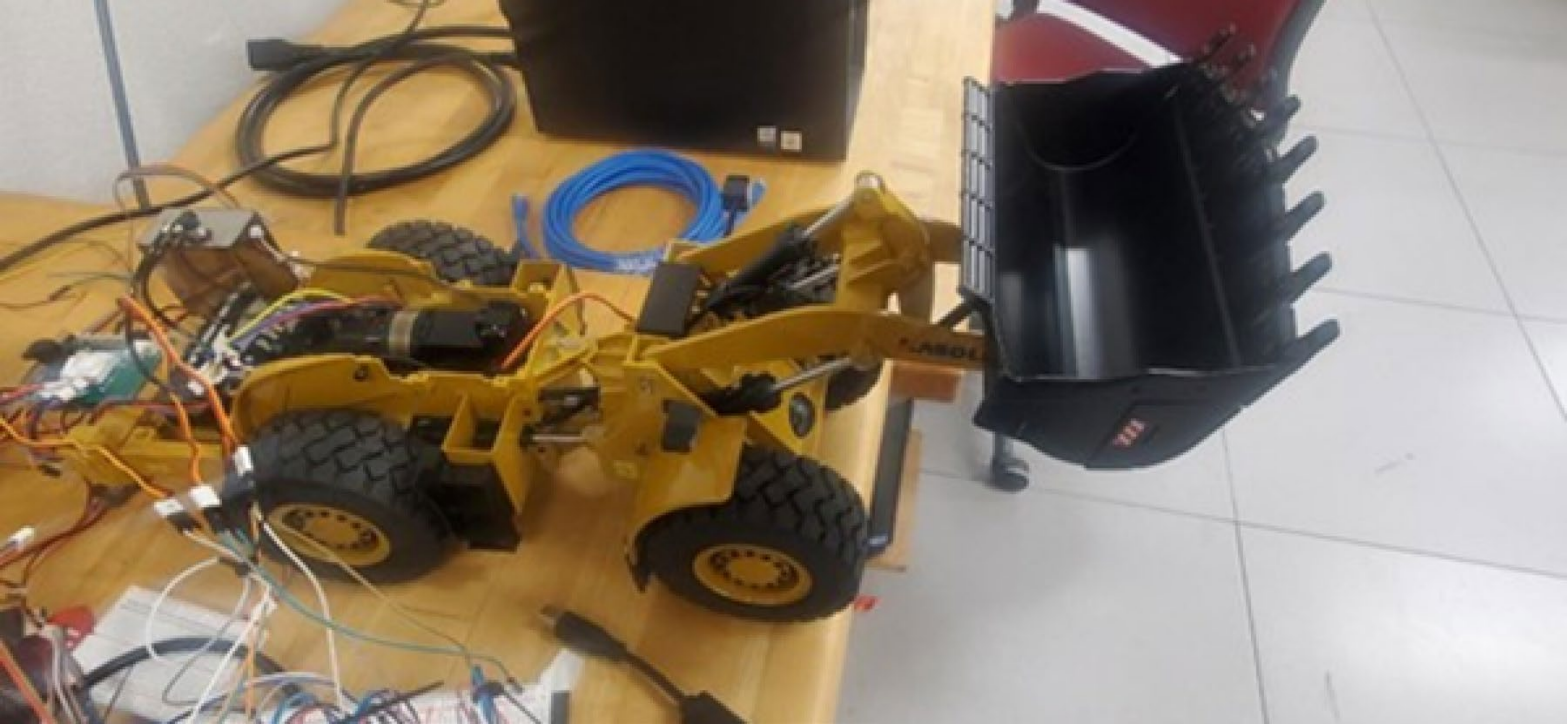
Miniature size wheel loader on a desk.

The trained scaling framework was validated on a Kabolite miniature wheel loader displayed in Fig. 10. Table 1 presents a comparison of the estimated masses of the end loader mechanism components of the EVERUN ER12 wheel loader and the miniature wheel loader. The estimations for the full-scale wheel loader were obtained from its detailed CAD model, which was constructed based on detailed measurements taken from the vehicle. The masses for the smaller wheel loader were estimated based on how the dimensions of the components scale.

**Table 1:** Masses of the end loader mechanism components for the full-size EVERUN ER12 wheel loader and the miniature wheel loader.

Part	Mass for the full-size wheel loader (kg)	Mass for the miniature wheel loader (kg)
Bucket	290.6	0.32
Link B	8.99	0.01
Link A	33.9	0.04
Main Arm	294.6	0.32
Hydraulic Rod A1	20.3	0.02
Hydraulic Rod A2	13.1	0.01
Hydraulic Rod B1	19.6	0.02
Hydraulic Rod B2	14.7	0.02

**Fig. 11:**
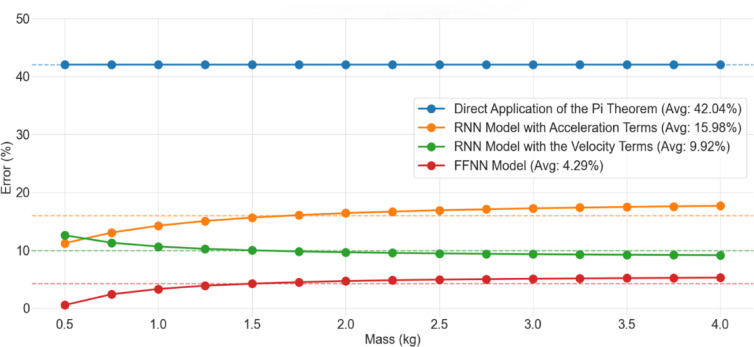
Errors in the scaling models’ estimations for the miniature wheel loader carrying known weights.

Unlike the EVERUN ER12 wheel loader, the miniature wheel loader does not feature an extensive set of sensors. Consequently, data for the miniature wheel loader were generated by assuming a static equilibrium condition under known weights, enabling an analytical solution for the required parameters. The model is exclusively trained using data from the Everun and Komatsu wheel loaders, while data from the miniature wheel loader are utilized for validation. The scaling models were evaluated under conditions where the load carried by the miniature wheel loader ranged from 0.5 to 4 kg. The upper limit was set to 4 kg by proportionally scaling down the maximum load capacity of the full-sized wheel loader relative to the overall vehicle weight of the miniature model. Figure 11 illustrates the evaluation of the scaling models on the miniature wheel loader, where the x-axis represents the load that the miniature wheel loader carries, while the y-axis represents the percentage error in the model’s estimations. Each curve corresponds to a different scaling model, and the dashed lines denote the average percentage error for each model across the entire load range. While Figs. 6 and 7 demonstrate the individual performances of the scaling models, Fig. 11 compares performance of all models in the same graph for the miniature wheel loader case against a baseline. To establish a baseline for comparison, the direct application of the Buckingham Pi theorem was employed. As anticipated, since this approach does not account for scaling factor distortions, it resulted in a significantly high average error of 42.04%. On the other hand, the best-performing scaling model was the FFNN-based model, which resulted in an average error of only 4.29%. This represents an approximate 90% reduction in error compared to the direct application of the Buckingham Pi theorem, which does not account for scaling distortions. This result demonstrates that it is feasible to use the scaling framework developed in this paper to reduce the number of sensors needed for DM calibration. In this context, leveraging scalability greatly reduces the cost of developing complex, physics-based DMs.

## Discussion

The results presented demonstrate that the proposed scaling framework was able to accurately use training data to estimate key parameters for systems of vastly different sizes, even in the presence of distorted scaling factors. Compared to previous approaches employing direct application of the Buckingham Pi theorem, the proposed framework can make accurate scaling estimations even when similitude requirements are not satisfied, such as the case illustrated with wheel loader DM calibration using a pre-existing product as a scaled model. Moreover, unlike the approaches utilizing regression-based models with dimensionless numbers to estimate parameter values, implementation developed here by using ML allowed accurate estimation of the target parameters in the presence of nonlinearity. The observed reduction in prediction error across multiple scales suggests that the method can reduce the number of sensors required for data collection across different scales within a single equipment product line, where critical parameters can be challenging to measure directly. From an industrial perspective, the primary benefit of employing the proposed ML-based scaling framework lies in its ability to reduce repeated instrumentation, calibration, and testing across product variants when similitude conditions are not satisfied. While scaled values of parameters can also be identified through expert knowledge, this process becomes increasingly challenging in the presence of non-uniform scaling factors across multiple scales. In such cases, expert-driven scaling must be repeatedly revisited and experimentally revalidated for each new configuration. This can lead to increased development times and costs. Once the ML-based framework presented here is trained, it enables rapid, repeatable, and consistent scaling of DMs across an entire product line, even under significant scaling distortions. In this context, ML serves as a force multiplier by capturing complex nonlinear scaling relationships that are difficult to derive analytically. Comparison of the model estimations and direct Pi theorem application on the miniature wheel loader illustrates that the FFNN-based model produced the most accurate results for the scaling process, and all three models greatly improved accuracy compared to the baseline direct Pi theorem application to the systems with distorted scaling factors.

## Conclusions

This paper presented the development of a scaling framework for DMs and validated its accuracy across wheel loaders of vastly different scales. The scaling framework integrated a novel methodology and modular computational programs that utilize DA and ML to facilitate the scaling process. The ability of the developed framework to account for the effects of distorted scaling factors on parameter scaling enables the use of small-scale units in similar production lines to be utilized in developing DMs of larger-scale units without the need for calibration equipment for larger-scale units. This would not be possible with the conventional scaling methods based solely on the Buckingham Pi theorem. The integration of ML was pivotal, particularly when it comes to capturing how distortions impact parameter scaling. The method developed can also be applied to other modeling domains beyond the wheel loader case study presented here, including complex systems where it is necessary to develop DMs of different scale units in the same product line.

While the present study validates the proposed scaling framework using digital models of wheel loaders across multiple scales, the broader applicability of the methodology for other systems has not yet been experimentally demonstrated. Extending the framework’s application to additional domains, such as other construction equipment or complex electromechanical systems, is a direction for future work. In such applications, the governing physical parameters, relevant dimensionless groups, and training datasets would need to be redefined according to the specific underlying physics of the system.

## Supplementary Information

Below is the link to the electronic supplementary material.

Supplementary Material 1

## Data Availability

All data supporting the findings of this study are available within the paper and its supplementary information files. Further supplementary materials are available from the corresponding author upon reasonable request.

## References

[1] Opoku, D. J., Perera, S., Osei-Kyei, R. & Rashidi, M. Digital twin application in the construction industry: A literature review. *J. Build. Eng.* **40**, 102726. 10.1016/j.jobe.2021.102726 (2021).

[2] Fu, T. et al. Digital twin-based excavation trajectory generation of uncrewed excavators for autonomous mining. *Autom. Constr.* **151**, 104855. 10.1016/j.autcon.2023.104855 (2023).

[3] You, K. et al. Earthwork digital twin for teleoperation of an automated bulldozer in edge dumping. *J. Field Robot.* **40**, 1945–1963. 10.1002/rob.22234 (2023).

[4] Talmaki, S. A. & Kamat, V. R. Sensor acquisition and allocation for real-time monitoring of articulated construction equipment in digital twins. *Sensors* **22**, 7635. 10.3390/s22197635 (2022). 36236733 10.3390/s22197635 PMC9571626

[5] Dadhich, S., Bodin, U. & Andersson, U. Key challenges in automation of earthmoving machines. *Autom. Constr.* **68**, 212–222. 10.1016/j.autcon.2016.05.009 (2016).

[6] Frank, B., Kleinert, J. & Filla, R. Optimal control of wheel loader actuators in gravel applications. *Autom. Constr.* **91**, 1–14. 10.1016/j.autcon.2018.03.005 (2018).

[7] Nezhadali, V., Frank, B. & Eriksson, L. Wheel loader operation—Optimal control compared to real drive experience. *Control Eng. Pract.* **48**, 1–9. 10.1016/j.conengprac.2015.12.015 (2016).

[8] Assadzadeh, A. et al. Excavator 3D pose estimation using deep learning and hybrid datasets. *Adv. Eng. Inform.* **55**, 101875. 10.1016/j.aei.2023.101875 (2023).

[9] Hussain, M., Ye, Z., Chi, H.-L. & Hsu, S.-C. Predicting degraded lifting capacity of aging tower cranes: A digital twin-driven approach. *Adv. Eng. Inform.* **59**, 102310. 10.1016/j.aei.2023.102310 (2024).

[10] Wu, B., Hou, L., Wang, S., Bu, X. & Xiang, C. Digital twin modeling for predicting loading resistance of loaders driven by deep transfer learning. *Adv. Eng. Inform.* **65**(Pt B), 103245. 10.1016/j.aei.2025.103245 (2025).

[11] Karanfil, D., Lindmark, D., Servin, M., Torick, D. & Ravani, B. Developing a calibrated physics-based digital twin for construction vehicles. *Digital Twin.*10.1080/27525783.2025.2592382 (2025).

[12] Murphy, G. *Similitude in Engineering* (Ronald Press Company, 1950).

[13] Grattan-Guinness, I. Joseph Fourier, *Théorie analytique de la chaleur* (1822). In *Grattan-Guinness, I (ed.) Landmark Writings in Western Mathematics 1640–1940,* 354–365 (Elsevier, 2005).

[14] Du, Y. et al. Scaling laws of space solar power satellite concentrator unit distortion model obtained by performance-driven separate similitude analysis method. *Aerosp. Sci. Technol.* **148**, 109081. 10.1016/j.ast.2024.109081 (2024).

[15] Rayleigh,. The principle of similitude. *Nature* **95**, 66–68. 10.1038/095066c0 (1915).

[16] Gibbings, J. C. *Dimensional Analysis* (Springer Science & Business Media, 2011).

[17] Szirtes, T. & Rózsa, P. *Applied Dimensional Analysis and Modeling* (Butterworth-Heinemann, 2011).

[18] Coutinho, C. P., Baptista, A. J. & Rodrigues, J. D. Reduced scale models based on similitude theory: A review up to 2015. *Eng. Struct.* **119**, 81–94. 10.1016/j.engstruct.2016.04.016 (2016).

[19] Buckingham, E. On physically similar systems; illustrations of the use of dimensional equations. *Phys. Rev.* **4**, 345–376. 10.1103/PhysRev.4.345 (1914).

[20] Tan, Q.-M. *Dimensional Analysis: With Case Studies in Mechanics* (Springer, 2011).

[21] Dumka, P., Chauhan, R., Singh, A., Singh, G. & Mishra, D. Implementation of Buckingham’s Pi theorem using Python. *Adv. Eng. Softw.* **173**, 103232. 10.1016/j.advengsoft.2022.103232 (2022).

[22] Constantine, P. G., del Rosario, Z. & Iaccarino, G. Data-driven dimensional analysis: algorithms for unique and relevant dimensionless groups. Preprint at https://arxiv.org/abs/1708.04303 (2017).

[23] Xie, X., Samaei, A., Guo, J., Liu, W. K. & Gan, Z. Data-driven discovery of dimensionless numbers and governing laws from scarce measurements. *Nat. Commun.* **13**, 7562. 10.1038/s41467-022-35084-w (2022). 36476735 10.1038/s41467-022-35084-w PMC9729234

[24] Zhao, J., Wang, F., Yu, B., Tong, P. & Chen, K. Experimental study on the ride comfort of a crawler power chassis scale model based on the similitude theory. *Chin. J. Mech. Eng.* **28**, 496–503. 10.3901/CJME.2015.0306.024 (2015).

[25] Davey, K., Darvizeh, R., Golbaf, A. & Sadeghi, H. The breaking of geometric similarity. *Int. J. Mech. Sci.* **187**, 105925. 10.1016/j.ijmecsci.2020.105925 (2020).

[26] Simitses, G. J., Starnes, J. & Rezaeepazhand, J. Structural similitude and scaling laws for plates and shells: a review. In *Advances in the Mechanics of Plates and Shells* (eds Durban, D., Givoli, D. & Simmonds, J. G.) *Solid Mechanics and its Applications,* vol. 88 (Springer, Dordrecht, 2001).

[27] Kasprzak, W., Lysik, B. & Rybaczuk, M. *Dimensional Analysis in the Identification of Mathematical Models*. (World Scientific, 1990).

[28] Zhou, S. et al. Physics-based machine learning method and the application to energy-consumption prediction in tunneling construction. *Adv. Eng. Inform.* **53**, 101642. 10.1016/j.aei.2022.101642 (2022).

[29] Mendez, P. F. & Ordóñez, F. Scaling laws from statistical data and dimensional analysis. *J. Appl. Mech.* **72**, 648–657. 10.1115/1.1943434 (2005).

[30] Zhou, Z., Qiu, C. & Zhang, Y. A comparative analysis of linear regression, neural networks and random forest regression for predicting air ozone employing soft sensor models. *Sci. Rep.* **13**, 22420. 10.1038/s41598-023-49899-0 (2023). 38104205 10.1038/s41598-023-49899-0 PMC10725498

[31] Song, Y., Wu, J., Liu, Z., Zhang, B. & Huang, T. Similitude analysis method of the dynamics of a hybrid spray-painting robot considering electromechanical coupling effect. *IEEE/ASME Trans. Mechatronics.* **26**, 2986–2997. 10.1109/TMECH.2021.3049388 (2021).

[32] Pearson, K. Mathematical contributions to the theory of evolution. III. Regression, heredity, and panmixia. *Philos. Trans. R. Soc. Lond. A* **187**, 253–318 (1896).

[33] Benesty, J., Chen, J. & Huang, Y. On the importance of the Pearson correlation coefficient in noise reduction. *IEEE Trans. Audio Speech Lang. Process.* **16**(4), 757–765. 10.1109/TASL.2008.919072 (2008).

[34] Terrero-Gonzalez, A., Dai, S., Neilson, R. D., Papadopoulos, J. & Kapitaniak, M. Dynamic response of a shallow-draft floating wind turbine concept: Experiments and modelling. *Renew. Energy* **226**, 120454. 10.1016/j.renene.2024.120454 (2024).

[35] Aoshima, K. & Servin, M. Examining the simulation-to-reality gap of a wheel loader digging in deformable terrain. *Multibody Syst. Dyn.* **64**, 121–148. 10.1007/s11044-024-10005-5 (2025).

[36] Abdolmohammadi, A., Mojahed, N., Nazari, S. & Ravani, B. Data-efficient excavation force estimation for wheel loaders. Preprint at https://arxiv.org/abs/2506.22579 (2025).

[37] Haas, M., Abdolmohammadi, A. & Nazari, S. Combined control and design optimization of a parallel electric-hydraulic hybrid wheel loader to prolong battery lifetime. Preprint at https://www.techrxiv.org/doi/full/10.36227/techrxiv.175423946.62231734 (2025).

[38] Svozil, D., Kvasnicka, V. & Pospichal, J. Introduction to multi-layer feed-forward neural networks. *Neurocomputing* **3**, 213–223. 10.1016/S0169-7439(97)00061-0 (1997).

[39] Panda, A., Ghosh, L. & Mahapatra, S. Integrating attention-based GRU with event-driven NMPC to enhance tracking performance of robotic manipulator under actuator failure. *Expert Syst. Appl.* **267**, 125946. 10.1016/j.eswa.2024.125946 (2025).

[40] Mienye, I. D., Swart, T. G. & Obaido, G. Recurrent neural networks: A comprehensive review of architectures, variants, and applications. *Info* **15**(9), 517. 10.3390/info15090517 (2024).

[41] Ogunsanya, M., Isichei, J. & Desai, S. Grid search hyperparameter tuning in additive manufacturing processes. *Manuf. Lett.* **35**(Suppl.), 1031–1042. 10.1016/j.mfglet.2023.08.056 (2023).

[42] Belete, D. M. & Huchaiah, M. D. Grid search in hyperparameter optimization of machine learning models for prediction of HIV/AIDS test results. *Int. J. Comput. Appl.* **44**(9), 875–886. 10.1080/1206212X.2021.1974663 (2021).

[43] Peng, C. Comprehensive analysis of the impact of learning rate and dropout rate on the performance of convolutional neural networks on the CIFAR-10 dataset. *Appl. Comput. Eng.* **102**, 183–192. 10.54254/2755-2721/102/20241161 (2024).

[44] Veerababu, D., Raikar, A. A. & Ghosh, P. K. Improving neural network training using dynamic learning rate schedule for PINNs and image classification. *Mach. Learn. Appl.* **21**, 100697. 10.1016/j.mlwa.2025.100697 (2025).

[45] Dhar, S. S. & Shalabh,. GIVE statistic for goodness of fit in instrumental variables models with application to COVID data. *Sci. Rep.* **12**, 9472. 10.1038/s41598-022-13240-y (2022). 35676510 10.1038/s41598-022-13240-y PMC9176169

[46] Raiaan, M. A. K. et al. A systematic review of hyperparameter optimization techniques in convolutional neural networks. *Decis. Anal. J.* **11**, 100470. 10.1016/j.dajour.2024.100470 (2024).

[47] Fakhrzad, F., ShariffAl-Sheikh, W. M., Mohammed, M. M. & Meftahizadeh, H. Machine learning models for predicting morphological traits and optimizing genotype and planting date in roselle (*Hibiscus sabdariffa* L.). *Sci. Rep.* **15**, 29148. 10.1038/s41598-025-15373-2 (2025). 40783447 10.1038/s41598-025-15373-2 PMC12335563

