# Supplementary material for: Scaling digital models
Source: Sci Rep. 2026 Jan 21;16:5962. doi: 10.1038/s41598-026-36310-x (PMC12902011; doi:10.1038/s41598-026-36310-x)
Supplement: Supplementary file 1 — Supplementary Material 1 [file 41598_2026_36310_MOESM1_ESM.docx]

Supplementary material

The dataset used for the paper[^48^](#ref48) and the supplementary demonstration video of the digital twin[^49^](#ref49) are given below. Additional supplementary material related to this study would be made available from the corresponding author upon reasonable request.

48. Karanfil, D. & Ravani, B. Scaling digital twin models data set. figshare <https://doi.org/10.6084/m9.figshare.29905919.v2> (2025).

49. Karanfil, D. Wheel loader digital twin media. figshare <https://doi.org/10.6084/m9.figshare.29868716.v1> (2025).
